# Effect of fungicidal contamination on survival, morphology, and cellular immunity of *Apis mellifera* (Hymenoptera: Apidae)

**DOI:** 10.3389/fphys.2023.1099806

**Published:** 2023-04-25

**Authors:** Gurleen Kaur, Amandeep Singh, Rohit Sharma, Abhinay Thakur, Shushant Tuteja, Shyamli, Randeep Singh

**Affiliations:** ^**1**^ PG Department of Agriculture, Khalsa College Amritsar, Amritsar, India; ^**2**^ Department of Agriculture, Khalsa College Garhdiwala, Hoshiarpur, India; ^**3**^ Department of Rasa Shastra and Bhaishajya Kalpana, Faculty of Ayurveda, Institute of Medical Sciences, Banaras Hindu University, Varanasi, India; ^4^ PG Department of Zoology, DAV College, Jalandhar, India

**Keywords:** honey bee, fungicide, granulocyte, larvae, morphology, survival

## Abstract

Pesticide residues have been reported in hive-stored products for long periods. Larvae of honey bees experience oral or contact exposure to these products during their normal growth and development inside the cells. We analyzed various toxicological, morphogenic, and immunological effects of residue-based concentrations of two fungicides, captan and difenoconazole, on the larvae of worker honey bees, *Apis mellifera*. Selected concentrations (0.08, 0.4, 2, 10, and 50 ppm) of both fungicides were applied topically at a volume of 1 µL/larva/cell as single and multiple exposures. Our results revealed a continuous, concentration-dependent decrease in brood survival after 24 h of treatment to the capping and emergence stages. Compared to larvae with a single exposure, the multiply exposed youngest larvae were most sensitive to fungicidal toxicity. The larvae that survived higher concentrations, especially multiple exposures, showed several morphological defects at the adult stage. Moreover, difenoconazole-treated larvae showed a significantly decreased number of granulocytes after 1 h of treatment followed by an increase after 24 h of treatment. Thus, fungicidal contamination poses a great risk as the tested concentrations showed adverse effects on the survival, morphology, and immunity of larval honey bees.

## Introduction

The economic and ecological importance of honey bees is high, as they provide valuable pollination services to crops, in addition to a variety of hive products (Rişcu and Bura, 2013). Biotic pollinators play an important role in food production and the maintenance of plant ecosystems; among these pollinators, bees are essential for good levels of pollination of most cultivated crops globally (Partap, 2011).

Since the 1990s, alarming declines in the populations of both wild and domesticated pollinators have been reported worldwide (Finley et al., 1996; Abrol, 2012). Interactions among different stressors like pests, diseases, pesticides, changing climate, and management practices are linked to these declines (Hristov et al., 2020). Moreover, race, comb age, cell size, and diet also affect the overall colony survival by inducing morphological or physiological variations (Abou-Shaara and Al-Ghamdi, 2012; Alfalah et al., 2012; Sauthier et al., 2017; Chole et al., 2019; Kaur et al., 2021). In addition, the geographic origin of a species and varying environmental conditions in a region also alter honey bee morphology (Al-Kahtani and Taha, 2014; Al-Kahtani and Taha, 2021; Shawer et al., 2021).

Among pesticides, insecticides cause maximum toxicity to bees, whereas other pesticides like fungicides, herbicides, and plant growth regulators are considered relatively safe (Mayer and Lunden, 1986; Devillers, 2002). However, recent studies have demonstrated the negative impact of fungicides on various hymenopterans (Heneberg et al., 2021; Thompson et al., 2023) including honey bees (Drummond, 2022; Serra et al., 2023; Xiong et al., 2023). The application of fungicides on blooming crops (Mayer and Lunden, 1986; Tamburini et al., 2021) leads to the direct contamination of forager bees, which, along with the collected pollen or nectar, transport fungicide residues to hives and, thus, contaminate the entire colony, including hive-stored products (Devillers, 2002; Bogdanov 2006). While the concentrations of fungicidal residues declined from treated flowers to forager bees (Drummond, 2022) to hive-stored honey and brood (Piechowicz et al., 2018), residues remain abundant in various hive products (Piechowicz et al., 2018; Rondeau and Raine, 2022; Xiao et al., 2022) and in some cases even exceeded the levels of concern for chronic risk to bees (Rondeau and Raine, 2022). The persistence of residues in hive-stored products may increase brood exposure to these residues. However, few studies have reported the role of residual fungicides on compromised bee health (Mullin et al., 2010; Rondeau and Raine, 2022). The toxicity criteria for field-recommended concentrations of captan toward honey bee brood and adults have been reported (Mussen et al., 2004; Ladurner et al., 2005) but are much higher than the residual forms of captan present in hive-stored products. Furthermore, fungicides like difenoconazole were studied only jointly with other pesticides or pests (Almasri et al., 2021; Pal et al., 2022), which masked their individual effects.

In the present scenario, the decline in the honey bee population is linked to weakened immune systems due to pesticides. These immuno-challenged bees are more prone to disease (Pamminger et al., 2018). Immunity in insects is generally comprised of cellular and humoral responses. At the cellular level, hemocytes (predominantly plasmatocytes and granulocytes) play an important role in immunity as they are involved in defense responses like phagocytosis, encapsulation, and nodulation, and show variations in their number in response to a foreign agent (Kwon et al., 2014; Negri et al., 2014; Barakat et al., 2016). The adverse effects of insecticides on the hemocytes of honey bees have been reported (Perveen and Ahmad, 2017; Sukkar et al., 2023) but similar studies for fungicides are lacking (Inoue et al., 2022). Until now no information has been reported regarding the adverse effects of residual forms of captan and difenoconazole on honey bee broods. Therefore, the present study assessed the individual effects of these fungicides (in both single and multiple exposures) on larvae survival and immunity and also on the morphology of adult bees developed from treated larvae.

## Materials and methods

### Hive selection

Three well-populated hives (each with 8–10 frames) of *A. mellifera* were selected from the apiary of Khalsa College, Amritsar (India) for each fungicide. These colonies were well maintained throughout the experimental period.

### Selection of fungicides and concentrations

Captan 50 WP (Captaf, Rallis India Ltd., Mumbai) and difenoconazole 25 EC (Score, Syngenta India Ltd., Pune) were selected for the study. The concentrations of both fungicides were selected based on reported residual amounts in various hive products. The maximum reported residues of difenoconazole in hive-stored honey, pollen, and bee bread varied from 0.0006–0.0009 mg/kg, 0.043–0.411 mg/kg, and 0.27–0.327 mg/kg, respectively. Similarly, the maximum reported residues of captan in hive-stored honey, pollen, bee wax, and bee bread varied from 0.009–0.019 mg/kg, 2.99–10.36 mg/kg, 0.069–0.4 mg/kg, and 6.39 mg/kg, respectively (Kubik et al., 2000; Bernal et al., 2010; Johnson et al., 2010; Mullin et al., 2010; Chauzat et al., 2011; Rennich et al., 2012; Stoner and Eitzer, 2013; McArt et al., 2017). An extensive literature review suggested that growing larvae may be exposed to a maximum of 10 ppm of captan and 0.4 ppm of difenoconazole either ectopically or via food. Therefore, the final concentrations (including the maximum reported residues and concentrations above and below these residues) of both fungicides were selected to be 0.08 ppm, 0.4 ppm, 2 ppm, 10 ppm, and 50 ppm. A negative control (NC) consisting of distilled water (solvent) and an untreated control (UC) were also included for comparison.

### Larval culture and maintenance to obtain uniformly aged worker larvae

To obtain uniformly aged larvae of worker honey bees, a vertical queen excluder was introduced in each colony to cage the queen on one side along with bees and an empty drawn frame for egg laying. After every 24 h, a frame with newly laid eggs was replaced with another empty comb for continuous egg laying. Frames with eggs were marked with the date of egg laying and kept on the other side of the hive. For the measurement of age, newly hatched larvae were defined as 1 day old and so on up to 6 days old. Accordingly, the larvae were divided into three age groups: 1–2, 3–4, and 5–6 days old.

### Application of fungicides

Different colored pins were used on a single frame to mark larvae receiving different concentrations and the controls. Depending upon the persistence of residues in hive products, risks related to honey bee health may increase due to increased exposure. The larval stage usually lasts for 5–6 days, and they are inspected and fed multiple times by multiple bees (Huang and Otis, 1991), which likely increases the possibility of larval exposure to contaminated food multiple times before the capping of their cells. Thus, in the present study, selected larvae were exposed both once and multiple times to selected concentrations of each fungicide. In one-time exposure (OTE), larvae belonging to the three different age groups were exposed once. In multiple exposures (ME), 1–2-day-old larvae were exposed four times, and 3–4-day-old larvae were exposed twice. From each tested concentration and solvent, 1 µL was delivered topically into cells containing larvae (Atkins and Kellum, 1986). A total of 90 larvae were selected (30 from each of three hives), for each concentration per age group per fungicide and control. After the treatments, the frames were returned to their respective hives, where they were fed by the nurse bees. Survival was checked 24 h after treatment (HAT), at capping, and at emergence.

### Morphometric analysis

**FIGURE 1:**
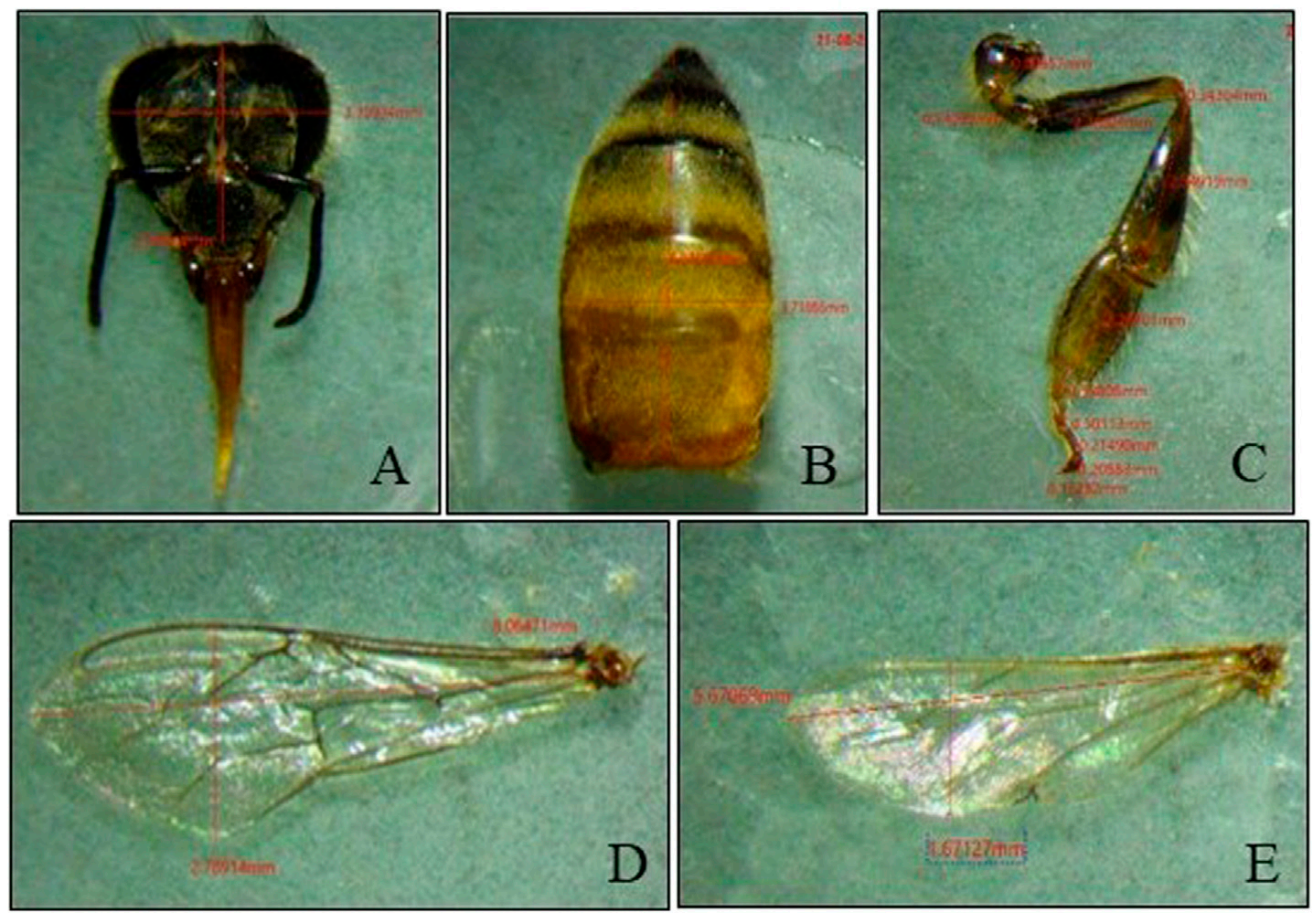
Measurement of **(A)** head, **(B)** abdomen, **(C)** hind leg, **(D)** forewing, and **(E)** hindwing sizes.

After capping, the treated larvae were caged and the emerging adults were collected and checked for morphological variations in length and width of the head; length and breadth of the abdomen, forewing, and hindwing; and length of the hind leg (Figure 1). All body parts were measured with a Magnus trinocular stereomicroscope (MSZ-TR [LED]) using MagVision advanced image analysis software (MIPS-ver 3.7).

### Cellular immunity analysis

Similar treatments were administered to 4-day-old worker larvae. Differential hemocyte counts (DHCs) were estimated at 1 HAT and 24 HAT. Hemolymph was extracted by puncturing the larva from the lateral side with a sterilized needle. Smears were prepared on clean glass slides. Cells were stained using Giemsa’s staining solution. The prepared slides were observed using a binocular microscope (Olympus CX21i LED) to identify cells based on the shape and size of the cells and nuclei, vacuolization, and cytoplasmic staining. A total of 100 cells per slide were counted, and three slides per treatment were observed containing hemolymph of three different larvae administered the same treatment.

### Statistical analysis

Statistical analyses were performed using IBM SPSS Statistics for Windows, version 21.0. One-way ANOVA followed by Tukey’s HSD (at a 0.05 level of significance) was used to analyze the effects of concentration on survival, morphology, and DHC. Two-way ANOVA was used to analyze the combined effect of concentration and time on DHC.

## Results

### Effects of captan and difenoconazole on worker larvae survival

**FIGURE 2:**
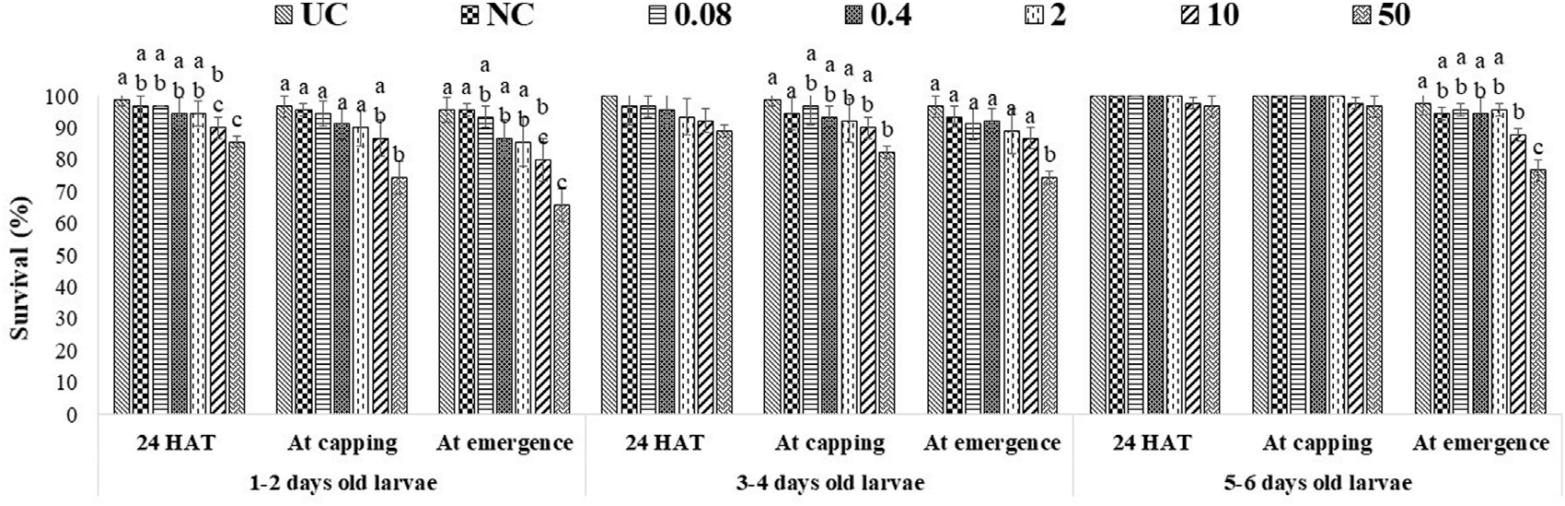
Survival of 1–2-, 3–4-, and 5–6-day-old honey bee larvae after OTE to captan. The different letters (a, b … ) indicate significant differences by Tukey’s HSD (*p* < 0.05).

**FIGURE 3:**
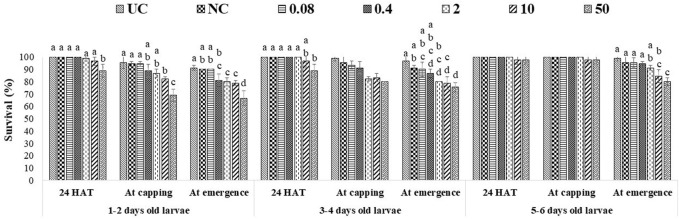
Survival of 1–2-, 3–4-, and 5–6-day-old honey bee larvae after OTE to difenoconazole The different letters (a, b … ) indicate significant differences by Tukey’s HSD (*p* < 0.05).

A single exposure to the 10 ppm concentration of captan significantly reduced the survival of 1–2-day-old larvae (at the emergence stage) compared to UC and NC. In addition, the 50 ppm concentration of captan significantly lowered the survival of 1–2-day-old larvae (at all stages of observation), 3–4-day-old larvae (capping and emergence stages), and 5–6-day-old larvae (emergence stage), with the lowest survival (65.7%) observed in 1–2-day-old larvae at the emergence stage (Figure 2). However, OTE to 10 and 50 ppm of difenoconazole significantly reduced the survival of 1–2-day-old larvae (at capping and emergence stage) and older 3–4- and 5–6-day-old larvae (at emergence stage), with the lowest survival (66.7%) observed in 1–2-day-old larvae at 50 ppm at the emergence stage (Figure 3). Moreover, 50 ppm of difenoconazole also lowered the survival of 1–2- and 3–4-day-old larvae after 24 h of treatment.

**FIGURE 4:**
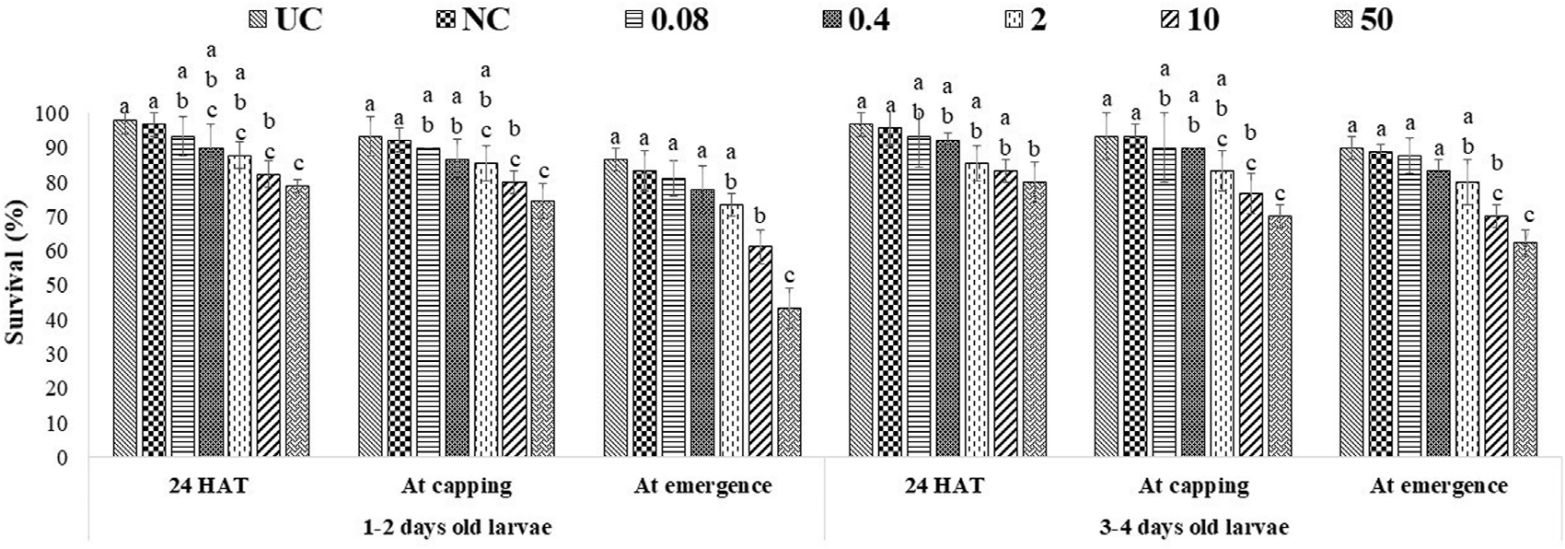
Survival of 1–2- and 3–4-day-old honey bee larvae after ME to captan. The different letters (a, b … ) indicate significant differences by Tukey’s HSD (*p* < 0.05).

**FIGURE 5:**
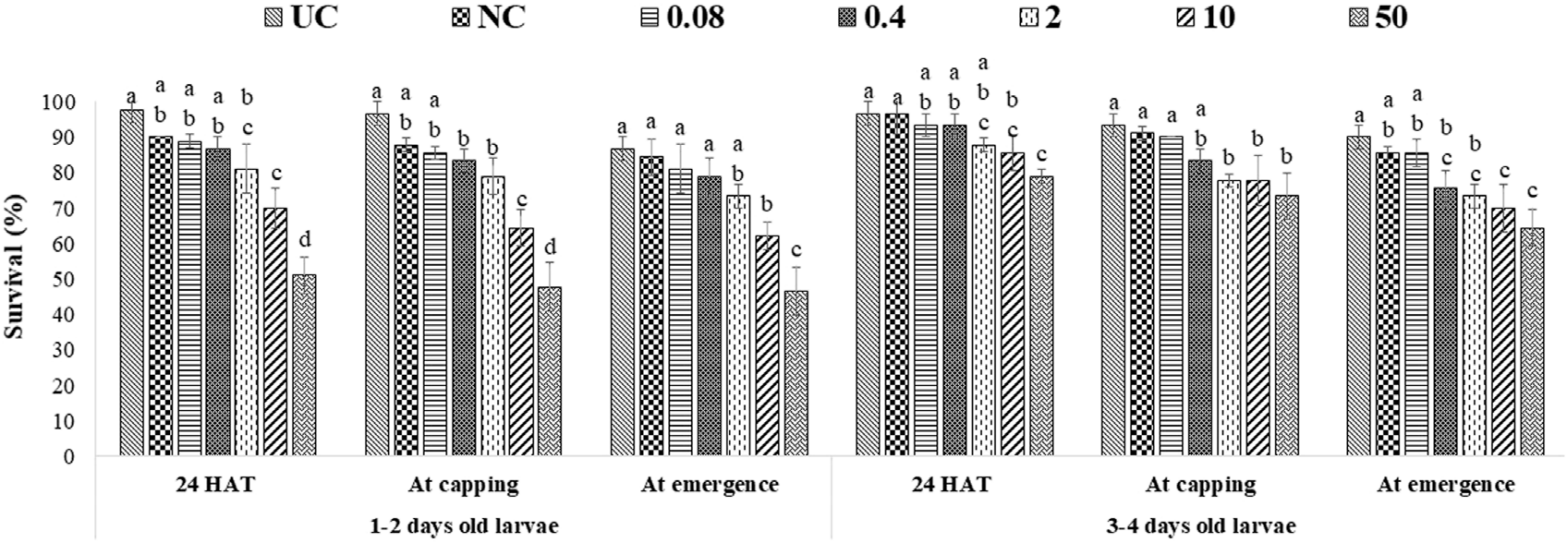
Survival of 1–2- and 3–4-day-old honey bee larvae after ME to difenoconazole. The different letters (a, b … ) indicate significant differences by Tukey’s HSD (*p* < 0.05).

ME had more pronounced effects, as 10 ppm and 50 ppm of both fungicides significantly lowered the survival of all tested age groups at all stages of observation. The lowest bee survival (44.3% for groups exposed to captan and 46.7% for groups exposed to difenoconazole) was observed at the emergence stage after exposing 1–2-day-old larvae to the 50 ppm concentration (Figures 4, 5). The lower tested concentrations had no significant effect on brood survival in both OTE and ME.

### Effects of captan and difenoconazole on the morphology of adults developed from treated worker larvae

**FIGURE 6:**
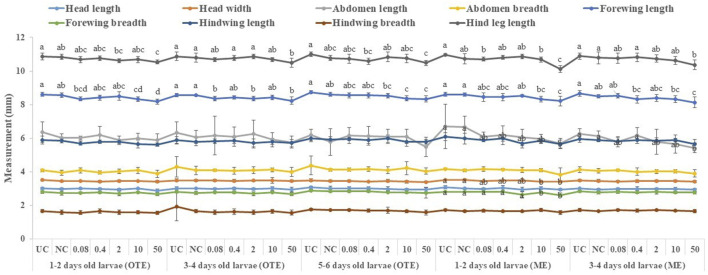
Morphological variations in adult honey bees developed from larvae after OTE and ME to captan. UC, untreated control; NC, negative control; OTE, one-time exposure; ME, multiple exposures. The different small letters (a, b … ) within a column indicate significant differences by Tukey’s HSD (*p* < 0.05).

**FIGURE 7:**
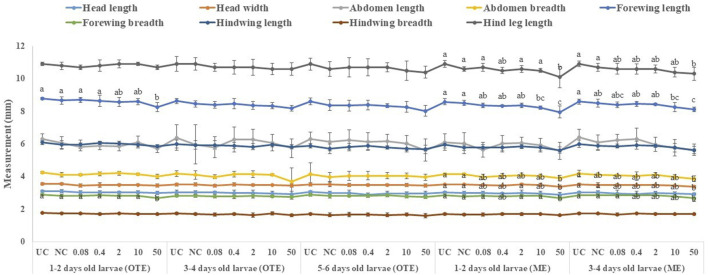
Morphological variations in adult honey bees developed from larvae after OTE and ME to difenoconazole. UC, untreated control; NC, negative control; OTE, one-time exposure; ME, multiple exposures. The different small letters (a, b … ) within a column indicate significant differences by Tukey’s HSD (*p* < 0.05).

All tested larval age groups after OTE to 10 and 50 ppm of captan showed significant reductions in forewing length at the adult stage compared to UC and NC, with maximum reductions at 50 ppm. Additionally, the hind leg length was also significantly reduced in bees developed from 1–2- and 3–4-day-old larvae after OTE to 50 ppm of captan (Figure 6). However, OTE to 50 ppm of difenoconazole significantly reduced the forewing length and breadth only in bees developed from 1–2-day-old larvae (Figure 7).

More significant variations were observed in ME than in OTE (Supplementary Table S1). The highest (50 ppm) concentration of captan significantly reduced the lengths of the abdomen, forewing, and hind leg in bees developed from both tested age groups of larvae. Additionally, bees developed from 1–2-day-old larvae treated with 10 and 50 ppm showed significantly reduced head width and forewing size (Figure 6). Head width, abdomen breadth, forewing length and breadth, and hind leg length were significantly reduced in bees exposed as 1–2-day-old larvae to 50 ppm of difenoconazole. Only the forewing length was significantly reduced at 10 ppm. Bees developed from 3–4-day-old treated larvae showed significant reductions in similar body parts except for the abdomen at 50 ppm (Figure 7).

### Effects of captan and difenoconazole on DHC of worker larvae

**FIGURE 8:**
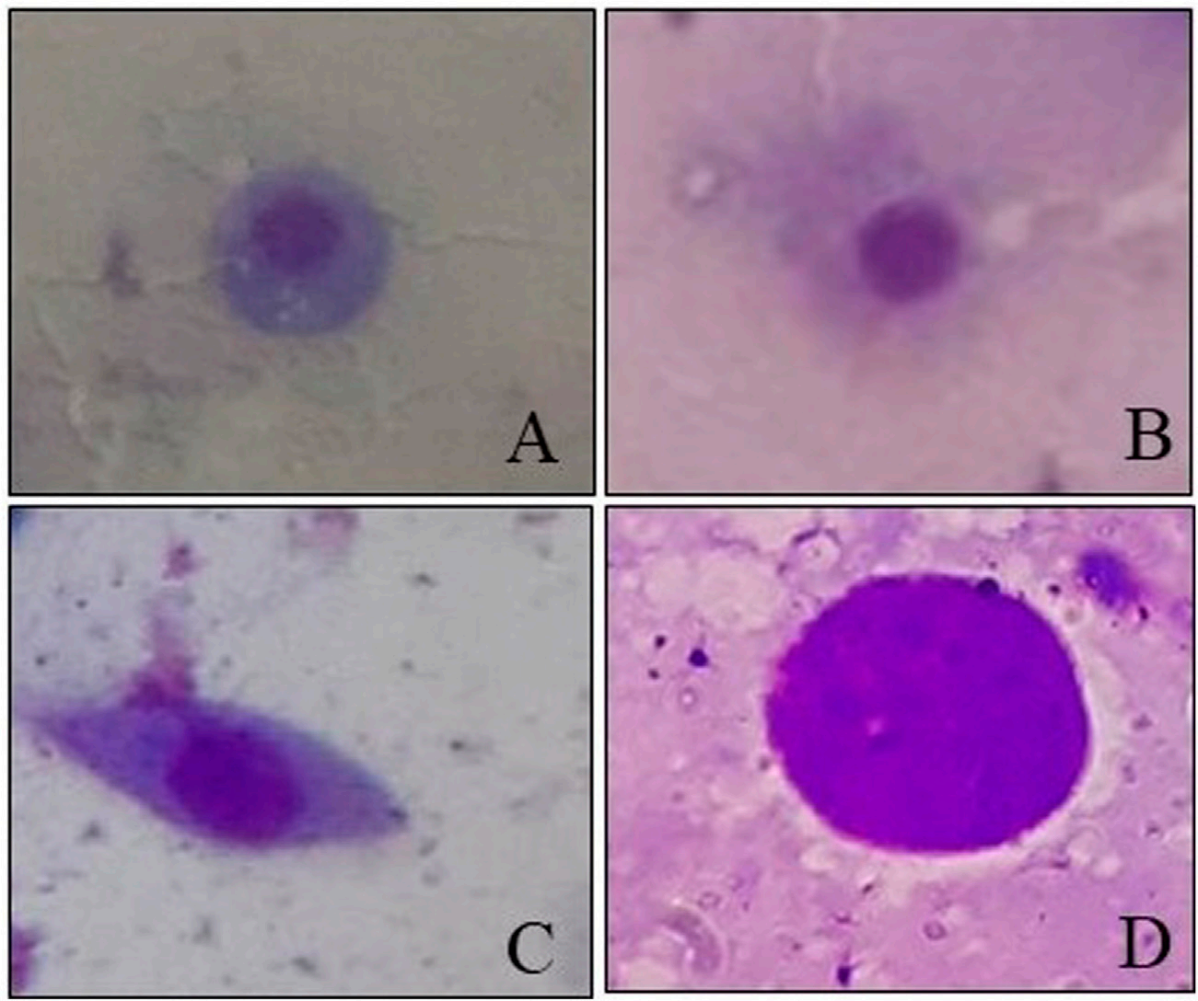
Hemocytes observed in worker honey bee larvae. **(A)** Granulocytes. **(B)** Permeabilized cell. **(C)** Spindle-shaped cell. **(D)** Permeable nucleus.

Four types of cells—granulocytes, permeabilized cells, spindle-shaped cells, and permeable nuclei—were identified from the hemolymph of 4-day-old worker larvae (Figure 8), with the dominance of granulocytes followed by permeabilized cells and other hemocyte communities (including spindle shaped-cells and permeable nuclei) in control larvae.

**FIGURE 9:**
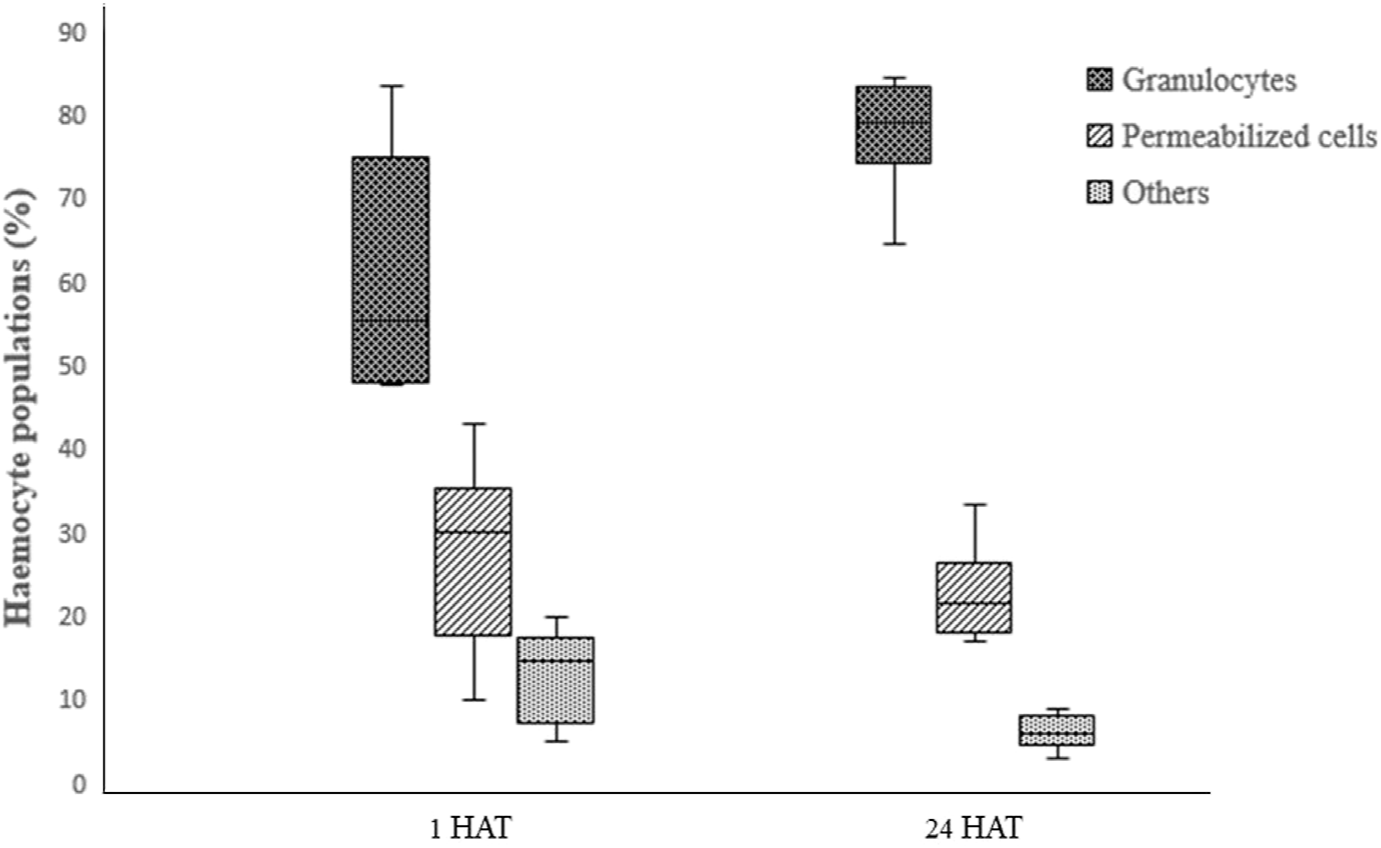
Effect of captan on the differential hemocyte counts (DHC) of honey bee larvae.

**FIGURE 10:**
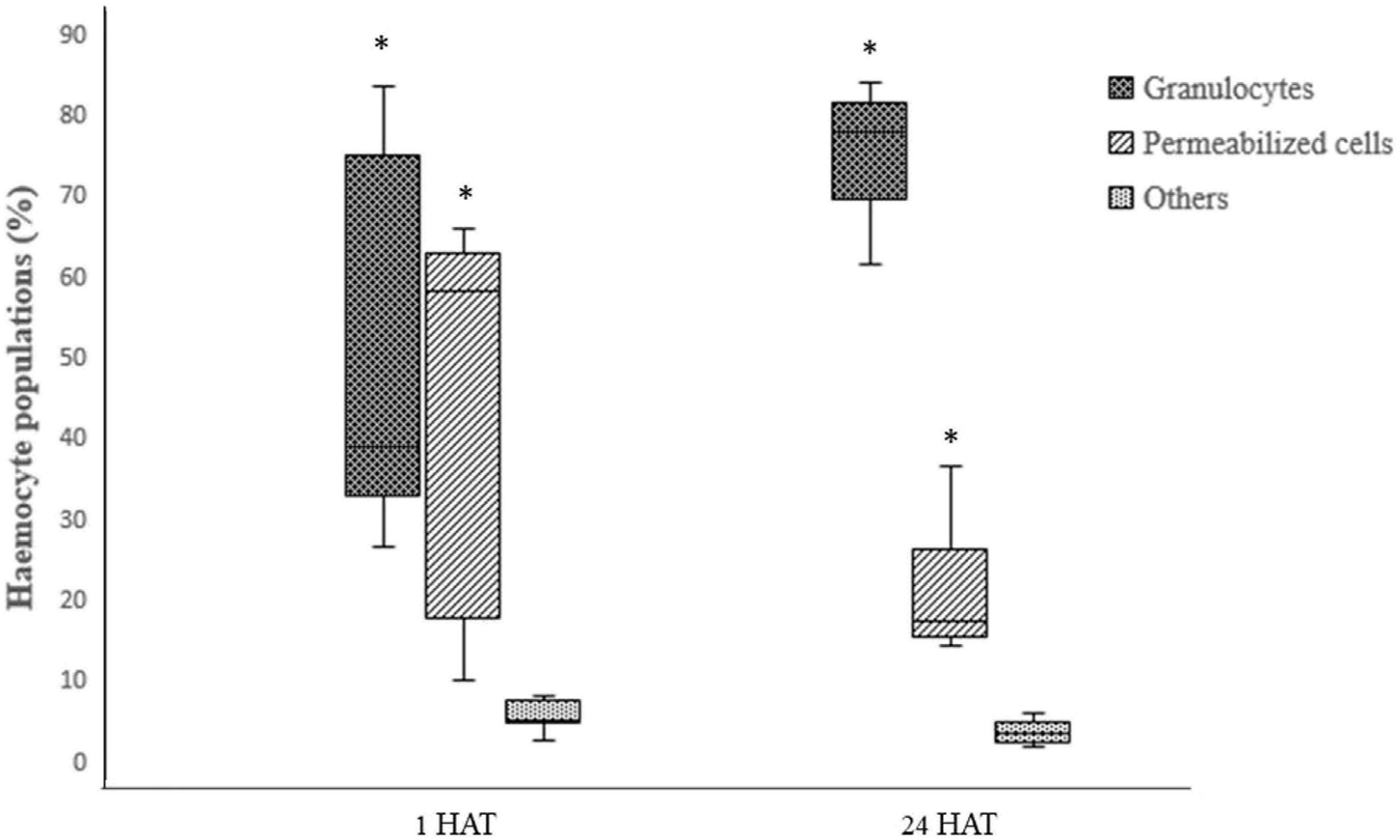
Effect of difenoconazole on the differential hemocyte counts (DHC) of honey bee larvae, where * indicates a significant difference by Tukey’s HSD (*p* < 0.05).

Two-way ANOVA analysis of captan-exposed larvae showed time-dependent but concentration-independent significant variations in hemocytes (Figure 9). However, two-way ANOVA analysis of difenoconazole-exposed larvae showed significant time- and concentration-dependent variations in hemocytes. Treated larvae at 1 HAT showed significant variations in the numbers of granulocytes and permeabilized cells at 10 and 50 ppm compared to UC and NC. The lowest granulocyte count, 26.3%, was observed at 50 ppm along with 65.7% permeabilized cells and 8% others. However, at 24 HAT, the counts were 61% granulocytes, 36.3% permeabilized cells, and 2.7% others, which was statistically non-significant compared to controls (Figure 10).

## Discussion

### Effects of fungicides on survival

Our results indicated that both tested fungicides had concentration- and age-dependent effects, with maximum damage at 50 ppm in the youngest (1–2-day-old) larvae followed by 3–4- and 5–6-day-old larvae. This age-dependent variation in sensitivity could have occurred due to two reasons. The first possibility is the increased body mass of older larvae. The average body weight of the oldest worker larva is more than 50 times that of the youngest larva (Malone et al., 2002). Since the amount of pesticide applied to any individual is a function of its body mass (Malbert-Colas et al., 2020), the quantity of fungicide applied per unit body mass of older larvae was lower compared to younger larvae, thus making older larvae less sensitive. Second, the age-specific effects of fungicides could be attributed to age-dependent variations in detoxifying enzyme levels, which usually increase with age in bees (Smirle, 1993). Detoxifying activity is further enhanced by phytochemicals like abscisic acid and *p*-coumaric acid, which are naturally present in bee food, i.e., pollen and honey (Mao et al., 2013; Negri et al., 2015). Food consumption usually increases with the age of the growing larva and peaks on the 4–5th day of larval age (Rortais et al., 2005). Furthermore, the quality of food consumed also varies with age as young larvae are predominantly fed royal jelly, while older larvae receive pollen (Haydak, 1970). Thus, the higher survival of older larvae may be due to greater consumption of pollen, which naturally contains phytochemicals responsible for pesticide tolerance and detoxification.

In our study, residue-based and higher concentrations of captan significantly altered brood survival. Captan shows antifungal activity due to its negative interaction with glutathione (Roberts et al., 2007), which also play an important role in oxidative stress management in animals (Wu et al., 2004). Thus, captan presumably has the same mode of action in honey bees as in fungi. Our results on *A. mellifera* agree with those of Mussen et al. (2004) who reported complete mortality of worker larvae after oral exposure to captan at a test dose of 0.8 mg/10 g diet (80 ppm/larva) but contrast with those of Everich et al*.* (2009) who reported nonsignificant effects of available commercial formulations of captan 50 WP and captan 80 WDH on overall hive health including foragers and brood after their application on blooming crops at a rate of 5 kg a. i./ha. As reported by Mussen et al. (2004), foragers are contaminated with 1 µg of pesticide for each 1 pound of pesticide applied per acre; thus, the application of 5 kg a. i./ha (4.46 lb/acre) in the study of Everich et al*.* (2009) would lead to the contamination of a visiting forager with 4.46 µg of captan (44.6 ppm/bee). These variations in the toxicity behavior of captan could be attributed to its different formulations, concentrations, exposure routes, and age or stage of the test insect.

We also found that higher concentrations of difenoconazole significantly reduced brood survival. Azole fungicide shows fungus toxicity due to its interference with cytochrome P-450 (Yoshida, 1988) which also regulates detoxification process in insects (Haas et al., 2022). Therefore, difenoconazole toxicity in honey bees occurs due to oxidation stress induced by the fungicide by reducing or inhibiting the activity of enzymes involved in antioxidant defenses (Pal et al., 2022).

### Effects of fungicides on morphology

Broods that survived higher fungicide concentrations showed various amorphogenic alterations in the adult stages. Our results indicated that residue-based concentrations of captan significantly reduced the sizes of the forewing and head in bees developed from the youngest group of larvae. Moreover, higher concentrations had more severe effects on morphology. These results agree with those of Atkins and Kellum. (1986), who showed that the negative effect of captan was greatly enhanced when applied to younger stages than later stages and that the contaminated larvae that survived developed into deformed bees. Our results also demonstrated that residue-based concentrations of difenoconazole had no adverse effect on the morphology of adults developed from treated larvae. However, higher concentrations significantly altered the morphology.

Morphological variations in adult insects may occur due to the disturbance of the imaginal discs of larvae (Gibson and Schubiger, 2000) which form the basic body structure in the adult stage, including legs in honey bees (Santos and Hartfelder, 2015). The treatment of larvae with fungicides may also disturb their imaginal discs; however, more research is needed on this topic. Other possible explanations for the morphogenic effects could be the altered behavior of nurse bees, as they change nursing activity with the condition of developing larvae (Siefert et al., 2020) and show lesser brood care in case of contaminated broods. The resulting malformed adults may interfere with the future work efficiencies of bees by reducing their lifespan (Wu et al., 2011), foraging (Higginson and Barnard, 2004), and learning activities (Worden et al., 2005).

### Effects of fungicides on DHC

The results of the current study provided experimental evidence that exposure of 4-day-old *A. mellifera* larvae to fungicides led to significant variations in DHC. Under normal conditions, we found that granulocytes are the most dominant hemocyte type, followed by permeabilized cells and others. Richardson et al. (2018) also reported similar types of cells with a predominance of granulocytes in worker honey bee larvae. Granulocytes and plasmatocytes are the two main types of hemocytes, which provide cellular immunity in insects through defense responses like encapsulation, phagocytosis, and nodulation. The other hemocyte components participate by interacting with these two hemocytes (Kwon et al., 2014).

The results of the current experiments demonstrated that with higher concentrations of difenoconazole, granulocyte numbers were significantly decreased while the counts of permeabilized cells and others were increased at 1 HAT. The decrease in granulocytes may have occurred due to reduced mitotic division or increased cell death following pesticide application (Uckan and Sak, 2010). Another possible explanation for these variations in granulocyte counts could be fungicide-generated oxidative stress (Han et al., 2016), which can cause cell injury (Sies, 2000). While the field-recommended concentrations of difenoconazole have very low or no toxicity to bees (Mommaerts and Smagghe, 2011), it enhances the toxicity of other pesticides when used in combination (Pal et al., 2022). This enhanced toxicity could be linked to the weakening of bee immune systems by difenoconazole due to reduced granulocyte count.

Furthermore, at 24 HAT, our results showed increases in granulocyte counts and decreases in all other hemocyte types. The increase in granulocytes might be due to the initiation of defense responses against fungicides. Our results are consistent with those reported by Negri et al. (2014) and Barakat et al. (2016), who also reported increased granulocyte counts in honey bee larvae at 24 HAT following exposure to other stress factors like nylon implants and bacteria injections. James and Xu (2012) reported that the increased total hemocyte and granulocyte counts were closely associated with cellular defense responses and detoxification of pesticides.

Both fungicides adversely affected the brood survival in a concentration-dependent manner. The effects of fungicides applied to immature stages (larvae) were observed in the mature stages as they developed into malformed adults. More pronounced effects of both tested fungicides were observed in multiply exposed larvae as compared to singly exposed larvae. The application of fungicides further affected larval immunity by reducing the number of granulocytes at 1 HAT. Therefore, fungicides, a class of pesticides generally considered to be relatively non-toxic to bees, showed noteworthy effects on the survival, morphology, and immunity of honey bee larvae. Thus, the toxicity criteria of fungicides must be re-evaluated, particularly in the context of immature honey bee stages.

## Data Availability

The original contributions presented in the study are included in the article/Supplementary Material. Further inquiries can be directed to the corresponding author.

## Abbreviations

ANOVA, analysis of variance; DHC, differential hemocyte count; Fig., figure; HAT, hours after treatment; HSD, honest significant difference; ME, multiple exposures; NC, negative control; OTE, one-time exposure; ppm, parts per million; mg/kg, milligrams per kilograms; lb/acre, pounds per acre; µg, microgram; µl, microliter; a.i./ha, active ingredient per hectare; %, percent, g, gram; mm, millimeter, UC, untreated control.
