# Supplementary material for: Effect of fungicidal contamination on survival, morphology, and cellular immunity of Apis mellifera (Hymenoptera: Apidae)
Source: Front Physiol. 2023 Apr 25;14:1099806. doi: 10.3389/fphys.2023.1099806 (PMC10167026; doi:10.3389/fphys.2023.1099806)
Supplement: Supplementary file 1 [file Table1.DOCX]

**Supplementary Table 1. Morphological variations in adult bees developed from larvae after OTE and ME to captan and difenoconazole**

| **Fungicide** | **Age group and Exposure** | **Conc. (ppm)** | **Head** | | **Abdomen** | | **Forewing** | | **Hindwing** | | **Hind Leg** |
| --- | --- | --- | --- | --- | --- | --- | --- | --- | --- | --- | --- |
|  |  |  | **Length (mm)** | **Width (mm)** | **Length (mm)** | **Breadth (mm)** | **Length (mm)** | **Breadth (mm)** | **Length (mm)** | **Breadth (mm)** | **Length (mm)** |
| **Captan** | **1-2 days old larvae (OTE)** | **UC** | 3.01^a^±0.06 | 3.51±0.06 | 6.36±0.62 | 4.08±0.09 | 8.61^a^±0.13 | 2.80±0.09 | 5.89^a^±0.24 | 1.67±0.08 | 10.87^a^±0.20 |
|  |  | **NC** | 2.98^ab^±0.06 | 3.44±0.05 | 6.03±0.22 | 3.95±0.15 | 8.56^ab^±0.13 | 2.73±0.13 | 5.84^ab^±0.11 | 1.60±0.13 | 10.84^ab^±0.16 |
|  |  | **0.08** | 3.00^a^±0.06 | 3.45±0.04 | 6.01±0.11 | 4.08±0.15 | 8.34^bcd^±0.11 | 2.75±0.05 | 5.70^ab^±0.12 | 1.56±0.12 | 10.69^abc^±0.18 |
|  |  | **0.4** | 2.98^ab^±0.07 | 3.42±0.07 | 6.21±0.48 | 3.97±0.10 | 8.42^abc^±0.16 | 2.77±0.07 | 5.80^ab^±0.10 | 1.66±0.14 | 10.76^abc^±0.14 |
|  |  | **2** | 2.95^ab^±0.08 | 3.46±0.06 | 5.90±0.22 | 4.04±0.14 | 8.51^abc^±0.25 | 2.70±0.13 | 5.78^ab^±0.15 | 1.58±0.14 | 10.62^bc^±0.11 |
|  |  | **10** | 3.00^a^±0.06 | 3.45±0.03 | 5.98±0.32 | 4.08±0.17 | 8.32^cd^±0.14 | 2.79±0.04 | 5.66^ab^±0.23 | 1.59±0.10 | 10.69^abc^±0.18 |
|  |  | **50** | 2.89^b^±0.10 | 3.43±0.07 | 5.89±0.27 | 3.89±0.22 | 8.18^d^±0.15 | 2.68±0.10 | 5.62^b^±0.10 | 1.55±0.11 | 10.54^c^±0.11 |
|  |  | **F-value** | **2.71*** | **1.77** | **1.99** | **2.20** | **7.84*** | **1.72** | **3.18*** | **1.26** | **4.67*** |
|  |  | **HSD** | **0.10** | **0.08** | **0.50** | **0.21** | **0.22** | **0.13** | **0.23** | **0.17** | **0.22** |
|  | **3-4 days old larvae (OTE)** | **UC** | 3.02±0.10 | 3.50±0.08 | 6.32±0.64 | 4.30±0.60 | 8.57^a^±0.10 | 2.80±0.06 | 5.88±0.21 | 1.94±0.85 | 10.88^a^±0.24 |
|  |  | **NC** | 3.01±0.04 | 3.50±0.07 | 6.07±0.38 | 4.09±0.25 | 8.56^a^±0.05 | 2.74±0.09 | 5.80±0.24 | 1.66±0.09 | 10.79^a^±0.27 |
|  |  | **0.08** | 2.99±0.06 | 3.48±0.07 | 6.16±1.14 | 4.11±0.07 | 8.36^b^±0.15 | 2.79±0.04 | 5.83±0.13 | 1.59±0.13 | 10.68^ab^±0.11 |
|  |  | **0.4** | 3.00±0.09 | 3.47±0.08 | 6.09±0.57 | 4.06±0.20 | 8.43^ab^±0.11 | 2.79±0.07 | 5.85±0.17 | 1.62±0.18 | 10.78^a^±0.14 |
|  |  | **2** | 2.97±0.07 | 3.48±0.05 | 6.26±0.83 | 4.09±0.21 | 8.35^b^±0.14 | 2.70±0.11 | 5.73±0.24 | 1.60±0.13 | 10.87^a^±0.14 |
|  |  | **10** | 3.01±0.12 | 3.49±0.18 | 5.91±0.43 | 4.12±0.11 | 8.44^ab^±0.13 | 2.78±0.05 | 5.80±0.19 | 1.65±0.12 | 10.69^ab^±0.12 |
|  |  | **50** | 2.93±0.16 | 3.45±0.06 | 5.76±0.36 | 3.99±0.28 | 8.24^b^±0.21 | 2.69±0.11 | 5.72±0.22 | 1.57±0.16 | 10.49^b^±0.27 |
|  |  | **F-value** | **0.75** | **0.21** | **0.74** | **0.92** | **6.81^*^** | **2.78** | **0.77** | **1.18** | **4.26*** |
|  |  | **HSD** | **0.14** | **0.13** | **0.93** | **0.41** | **0.18** | **0.11** | **0.28** | **0.48** | **0.27** |
|  | **5-6 days old larvae (OTE)** | **UC** | 3.07±0.09 | 3.50^a^±0.08 | 6.21±0.34 | 4.38±0.56 | 8.75^a^±0.10 | 2.88±0.05 | 5.98±0.20 | 1.77^a^±0.07 | 11.00^a^±0.14 |
|  |  | **NC** | 3.01±0.14 | 3.45^ab^±0.07 | 5.78±0.80 | 4.12±0.08 | 8.61^ab^±0.12 | 2.86±0.08 | 5.93±0.16 | 1.72^ab^±0.04 | 10.78^abc^±0.16 |
|  |  | **0.08** | 3.02±0.10 | 3.44^ab^±0.07 | 6.15±0.47 | 4.14±0.10 | 8.55^abc^±0.16 | 2.83±0.06 | 5.95±0.10 | 1.72^ab^±0.07 | 10.73^abc^±0.26 |
|  |  | **0.4** | 3.03±0.08 | 3.43^ab^±0.04 | 6.14±0.55 | 4.16±0.13 | 8.56^abc^±0.15 | 2.83±0.07 | 5.90±0.22 | 1.69^ab^±0.09 | 10.58^bc^±0.19 |
|  |  | **2** | 2.99±0.12 | 3.47^ab^±0.10 | 6.11±0.41 | 4.09±0.20 | 8.53^bc^±0.15 | 2.79±0.06 | 5.98±0.21 | 1.70^ab^±0.18 | 10.84^ab^±0.28 |
|  |  | **10** | 2.95±0.08 | 3.41^ab^±0.08 | 6.11±0.42 | 4.22±0.38 | 8.37^c^±0.16 | 2.79±0.09 | 5.80±0.27 | 1.65^ab^±0.09 | 10.75^abc^±0.24 |
|  |  | **50** | 2.94±0.13 | 3.39^b^±0.05 | 5.48±0.58 | 4.04±0.19 | 8.34^c^±0.19 | 2.75±0.31 | 5.80±0.13 | 1.58^b^±0.15 | 10.48^c^±0.16 |
|  |  | **F-value** | **1.41** | **2.25*** | **2.30** | **1.32** | **7.26*** | **0.93** | **1.43** | **2.59*** | **5.66*** |
|  |  | **HSD** | **0.15** | **0.10** | **0.73** | **0.39** | **0.21** | **0.19** | **0.27** | **0.15** | **0.29** |
|  | **1-2 days old larvae (ME)** | **UC** | 3.08±0.09 | 3.53^a^±0.04 | 6.70^a^±1.32 | 4.18^a^±0.05 | 8.61^a^±0.11 | 2.81^a^±0.03 | 6.10^a^±0.23 | 1.73^a^±0.05 | 10.97^a^±0.06 |
|  |  | **NC** | 3.01±0.05 | 3.52^a^±0.06 | 6.66^a^±0.64 | 4.09^ab^±0.09 | 8.60^a^±0.09 | 2.80^a^±0.04 | 5.98^ab^±0.31 | 1.67^ab^±0.07 | 10.73^ab^±0.29 |
|  |  | **0.08** | 2.98±0.06 | 3.44^ab^±0.06 | 6.11^ab^±0.27 | 4.17^a^±0.13 | 8.45^abc^±0.25 | 2.81^a^±0.05 | 5.90^ab^±0.11 | 1.68^ab^±0.08 | 10.70^b^±0.12 |
|  |  | **0.4** | 3.05±0.13 | 3.49^ab^±0.04 | 6.19^ab^±0.56 | 4.14^a^±0.05 | 8.45^abc^±0.21 | 2.81^a^±0.05 | 5.98^ab^±0.17 | 1.65^ab^±0.05 | 10.81^ab^±0.11 |
|  |  | **2** | 2.96±0.12 | 3.50^ab^±0.09 | 6.05^ab^±0.49 | 4.10^ab^±0.15 | 8.53^ab^±0.08 | 2.78^a^±0.05 | 5.69^b^±0.27 | 1.65^ab^±0.06 | 10.87^ab^±0.13 |
|  |  | **10** | 3.02±0.08 | 3.43^b^±0.07 | 5.97^ab^±0.27 | 4.09^ab^±0.11 | 8.32^bc^±0.17 | 2.63^b^±0.17 | 5.87^ab^±0.16 | 1.72^a^±0.11 | 10.68^b^±0.15 |
|  |  | **50** | 2.95±0.06 | 3.43^b^±0.07 | 5.69^b^±0.30 | 3.84^b^±0.39 | 8.21^c^±0.30 | 2.62^b^±0.11 | 5.66^b^±0.23 | 1.58^b^±0.12 | 10.13^c^±0.21 |
|  |  | **F-value** | **2.24** | **4.26^*^** | **2.82*** | **3.65*** | **4.98*** | **8.40*** | **4.40*** | **2.93*** | **22.02*** |
|  |  | **HSD** | **0.13** | **0.08** | **0.90** | **0.24** | **0.26** | **0.12** | **0.31** | **0.11** | **0.24** |
|  | **3-4 days old larvae (ME)** | **UC** | 3.03±0.03 | 3.49±0.07 | 6.27^a^±0.30 | 4.13±0.18 | 8.68^a^±0.17 | 2.84±0.07 | 5.95^a^±0.21 | 1.73±0.09 | 10.89^a^±0.19 |
|  |  | **NC** | 2.93±0.09 | 3.47±0.08 | 6.13^a^±0.30 | 4.06±0.13 | 8.50^ab^±0.11 | 2.77±0.09 | 5.90^ab^±0.12 | 1.67±0.03 | 10.79^a^±0.35 |
|  |  | **0.08** | 2.97±0.08 | 3.43±0.06 | 5.79^ab^±0.30 | 4.09±0.07 | 8.53^ab^±0.12 | 2.80±0.10 | 5.83^ab^±0.18 | 1.73±0.06 | 10.76^ab^±0.30 |
|  |  | **0.4** | 2.99±0.11 | 3.44±0.08 | 6.17^a^±0.47 | 4.00±0.22 | 8.32^bc^±0.22 | 2.79±0.09 | 5.89^ab^±0.21 | 1.69±0.10 | 10.82^a^±0.26 |
|  |  | **2** | 2.97±0.07 | 3.44±0.07 | 5.79^ab^±0.76 | 4.02±0.12 | 8.39^abc^±0.21 | 2.82±0.14 | 5.87^ab^±0.17 | 1.72±0.05 | 10.74^ab^±0.22 |
|  |  | **10** | 2.99±0.09 | 3.45±0.06 | 5.66^ab^±0.55 | 4.03±0.09 | 8.33^bc^±0.19 | 2.77±0.07 | 5.90^ab^±0.10 | 1.68±0.06 | 10.63^ab^±0.25 |
|  |  | **50** | 2.93±0.09 | 3.42±0.06 | 5.43^b^±0.29 | 3.91±0.23 | 8.14^c^±0.32 | 2.76±0.07 | 5.65^b^±0.26 | 1.65±0.10 | 10.37^b^±0.29 |
|  |  | **F-value** | **1.53** | **0.95** | **3.99*** | **1.65** | **6.46*** | **0.76** | **2.43*** | **1.47** | **3.47*** |
|  |  | **HSD** | **0.12** | **0.09** | **0.63** | **0.22** | **0.28** | **0.13** | **0.26** | **0.11** | **0.38** |
| **Difenoconazole** | **1-2 days old larvae (OTE)** | **UC** | 3.10±0.06 | 3.55±0.06 | 6.29^a^±0.32 | 4.24^a^±0.07 | 8.79^a^±0.07 | 2.87^a^±0.07 | 6.08±0.12 | 1.77^a^±0.04 | 10.96±0.11 |
|  |  | **NC** | 3.10±0.10 | 3.54±0.06 | 6.05^ab^±0.38 | 4.10^ab^±0.15 | 8.66^a^±0.22 | 2.83^a^±0.09 | 5.95±0.25 | 1.74^ab^±0.06 | 10.88±0.23 |
|  |  | **0.08** | 3.03±0.10 | 3.45±0.11 | 5.81^b^±0.25 | 4.12^ab^±0.07 | 8.70^a^±0.16 | 2.82^a^±0.05 | 5.96±0.26 | 1.72^ab^±0.05 | 10.78±0.15 |
|  |  | **0.4** | 3.02±0.10 | 3.47±0.14 | 5.89^ab^±0.25 | 4.16^a^±0.11 | 8.64^a^±0.35 | 2.85^a^±0.11 | 6.05±0.11 | 1.71^ab^±0.07 | 10.83±0.34 |
|  |  | **2** | 3.03±0.08 | 3.49±0.06 | 5.86^ab^±0.30 | 4.20^a^±0.12 | 8.57^ab^±0.29 | 2.81^a^±0.08 | 6.04±0.12 | 1.74^ab^±0.04 | 10.95±0.24 |
|  |  | **10** | 3.04±0.09 | 3.49±0.06 | 6.10^ab^±0.37 | 4.13^ab^±0.07 | 8.59^ab^±0.18 | 2.81^a^±0.05 | 5.97±0.17 | 1.71^ab^±0.05 | 10.92±0.10 |
|  |  | **50** | 2.99±0.10 | 3.44±0.09 | 5.72^b^±0.24 | 4.00^b^±0.14 | 8.26^b^±0.28 | 2.67^b^±0.11 | 5.84±0.11 | 1.68^b^±0.06 | 10.74±0.15 |
|  |  | **F-value** | **1.68** | **1.85** | **3.62*** | **4.43*** | **4.22*** | **4.75*** | **1.78** | **2.18*** | **1.50** |
|  |  | **HSD** | **0.13** | **0.12** | **0.43** | **0.15** | **0.33** | **0.12** | **0.24** | **0.07** | **0.28** |
|  | **3-4 days old larvae (OTE)** | **UC** | 3.02±0.14 | 3.50±0.09 | 6.39±0.79 | 4.18±0.17 | 8.63^a^±0.15 | 2.83±0.08 | 6.00±0.25 | 1.73±0.07 | 10.94±0.45 |
|  |  | **NC** | 3.02±0.12 | 3.50±0.11 | 5.96±1.20 | 4.09±0.27 | 8.46^ab^±0.19 | 2.82±0.11 | 5.93±0.27 | 1.70±0.08 | 10.92±0.39 |
|  |  | **0.08** | 3.02±0.07 | 3.43±0.09 | 5.79±0.65 | 3.98±0.12 | 8.40^ab^±0.20 | 2.77±0.11 | 5.93±0.26 | 1.66±0.09 | 10.70±0.26 |
|  |  | **0.4** | 3.00±0.16 | 3.50±0.11 | 6.26±0.76 | 4.13±0.18 | 8.47^ab^±0.32 | 2.78±0.15 | 5.90±0.19 | 1.70±0.07 | 10.73±0.43 |
|  |  | **2** | 3.00±0.15 | 3.47±0.08 | 6.27±0.62 | 4.14±0.21 | 8.36^ab^±0.24 | 2.81±0.12 | 5.83±0.20 | 1.63±0.13 | 10.78±0.48 |
|  |  | **10** | 2.97±0.13 | 3.48±0.04 | 6.07±0.50 | 4.10±0.08 | 8.34^ab^±0.19 | 2.78±0.07 | 5.94±0.14 | 1.73±0.11 | 10.68±0.34 |
|  |  | **50** | 2.92±0.16 | 3.45±0.11 | 5.73±0.58 | 3.69±0.85 | 8.18^b^±0.18 | 2.73±0.11 | 5.83±0.11 | 1.62±0.09 | 10.67±0.39 |
|  |  | **F-value** | **0.61** | **0.74** | **0.99** | **1.91** | **3.47^*^** | **0.72** | **0.75** | **1.74** | **0.69** |
|  |  | **HSD** | **0.19** | **0.13** | **1.05** | **0.50** | **0.30** | **0.16** | **0.29** | **0.13** | **0.55** |
|  | **5-6 days old larvae (OTE)** | **UC** | 3.06±0.07 | 3.52±0.11 | 6.31±0.42 | 4.14±0.68 | 8.60^a^±0.21 | 2.87±0.08 | 5.89±0.14 | 1.71±0.05 | 10.92±0.36 |
|  |  | **NC** | 2.98±0.11 | 3.51±0.15 | 6.13±0.57 | 3.96±0.27 | 8.37^ab^±0.33 | 2.81±0.14 | 5.70±0.29 | 1.64±0.13 | 10.67±0.46 |
|  |  | **0.08** | 2.99±0.16 | 3.48±0.11 | 6.25±0.62 | 4.04±0.28 | 8.35^ab^±0.34 | 2.83±0.09 | 5.80±0.22 | 1.67±0.14 | 10.77±0.59 |
|  |  | **0.4** | 2.90±0.09 | 3.46±0.07 | 6.14±0.55 | 4.05±0.25 | 8.38^ab^±0.30 | 2.82±0.10 | 5.88±0.23 | 1.66±0.10 | 10.71±0.51 |
|  |  | **2** | 2.96±0.12 | 3.49±0.07 | 6.18±0.41 | 4.05±0.19 | 8.34^ab^±0.13 | 2.84±0.08 | 5.78±0.12 | 1.64±0.11 | 10.77±0.29 |
|  |  | **10** | 2.97±0.09 | 3.48±0.10 | 6.04±0.48 | 4.03±0.17 | 8.27^ab^±0.32 | 2.79±0.10 | 5.72±0.23 | 1.67±0.08 | 10.52±0.59 |
|  |  | **50** | 2.95±0.15 | 3.45±0.11 | 5.62±0.68 | 3.95±0.18 | 8.03^b^±0.33 | 2.75±0.10 | 5.66±0.27 | 1.59±0.13 | 10.41±0.36 |
|  |  | **F-value** | **1.44** | **0.37** | **1.56** | **0.32** | **2.91*** | **1.18** | **1.38** | **0.90** | **1.17** |
|  |  | **HSD** | **0.17** | **0.15** | **0.74** | **0.46** | **0.40** | **0.14** | **0.31** | **0.15** | **0.65** |
|  | **1-2 days old larvae (ME)** | **UC** | 3.03±0.07 | 3.50^a^±0.02 | 6.11±0.50 | 4.14^a^±0.08 | 8.58^a^±0.20 | 2.86^a^±0.04 | 5.95^a^±0.19 | 1.69±0.07 | 10.90^a^±0.21 |
|  |  | **NC** | 2.99±0.12 | 3.50^a^±0.06 | 6.04±0.66 | 4.13^a^±0.09 | 8.51^a^±0.16 | 2.79^a^±0.07 | 5.77^ab^±0.15 | 1.66±0.06 | 10.60^a^±0.14 |
|  |  | **0.08** | 2.99±0.09 | 3.46^ab^±0.07 | 5.64±0.46 | 3.98^ab^±0.16 | 8.37^ab^±0.14 | 2.82^a^±0.04 | 5.78^ab^±0.18 | 1.66±0.10 | 10.72^a^±0.25 |
|  |  | **0.4** | 2.97±0.06 | 3.45^ab^±0.03 | 6.03±0.47 | 4.04^ab^±0.15 | 8.33^ab^±0.05 | 2.79^ab^±0.06 | 5.77^ab^±.026 | 1.68±0.07 | 10.55^ab^±0.19 |
|  |  | **2** | 2.99±0.16 | 3.50^a^±0.09 | 6.07±0.34 | 4.08^ab^±0.13 | 8.36^ab^±0.13 | 2.82^a^±0.06 | 5.84^ab^±0.13 | 1.68±0.09 | 10.68^a^±0.20 |
|  |  | **10** | 2.96±0.12 | 3.47^ab^±0.10 | 5.93±0.42 | 4.02^ab^±0.13 | 8.21^bc^±0.11 | 2.81^a^±0.06 | 5.77^ab^±0.20 | 1.68±0.03 | 10.59^a^±0.12 |
|  |  | **50** | 2.90±0.12 | 3.38^b^±0.11 | 5.58±0.53 | 3.89^b^±0.18 | 7.96^c^±0.38 | 2.68^b^±0.12 | 5.62^b^±0.18 | 1.61±0.06 | 10.11^b^±0.65 |
|  |  | **F-value** | **1.08** | **2.40*** | **1.68** | **3.55*** | **9.62*** | **5.15*** | **2.44*** | **1.03** | **5.71*** |
|  |  | **HSD** | **0.15** | **0.10** | **0.68** | **0.19** | **0.27** | **0.10** | **0.26** | **0.10** | **0.42** |
|  | **3-4 days old larvae (ME)** | **UC** | 3.04±0.07 | 3.51^a^±0.08 | 6.41^a^±0.49 | 4.18^a^±0.20 | 8.60^a^±0.16 | 2.85^a^±0.04 | 6.00^a^±0.23 | 1.74±0.07 | 10.90^a^±0.18 |
|  |  | **NC** | 3.03±0.10 | 3.49^a^±0.14 | 6.09^ab^±0.47 | 4.11^ab^±0.11 | 8.50^ab^±0.25 | 2.85^a^±0.10 | 5.89^ab^±0.25 | 1.72±0.05 | 10.78^a^±0.26 |
|  |  | **0.08** | 2.95±0.22 | 3.46^ab^±0.09 | 6.22^ab^±0.36 | 4.06^ab^±0.09 | 8.40^abc^±0.18 | 2.85^a^±0.08 | 5.86^ab^±0.13 | 1.68±0.05 | 10.69^ab^±0.33 |
|  |  | **0.4** | 2.92±0.18 | 3.47^ab^±0.04 | 6.32^ab^±0.63 | 4.02^ab^±0.25 | 8.46^ab^±0.16 | 2.84^ab^±0.06 | 5.92^ab^±0.23 | 1.73±0.05 | 10.61^ab^±0.32 |
|  |  | **2** | 2.99±0.11 | 3.48^ab^±0.03 | 5.97^ab^±0.44 | 4.09^ab^±0.16 | 8.44^ab^±0.07 | 2.84^ab^±0.04 | 5.88^ab^±0.10 | 1.71±0.05 | 10.69^ab^±0.25 |
|  |  | **10** | 2.95±0.10 | 3.43^ab^±0.05 | 5.75^ab^±0.51 | 3.98^ab^±0.12 | 8.26^bc^±0.28 | 2.77^ab^±0.08 | 5.77^ab^±0.27 | 1.69±0.05 | 10.47^ab^±0.31 |
|  |  | **50** | 2.92±0.08 | 3.36^b^±0.07 | 5.65^b^±0.35 | 3.87^b^±0.17 | 8.12^c^±0.13 | 2.69^b^±0.23 | 5.62^b^±0.23 | 1.67±0.08 | 10.34^b^±0.39 |
|  |  | **F-value** | **1.20** | **2.92*** | **3.18*** | **2.92*** | **6.09*** | **2.74*** | **2.81*** | **1.56** | **3.56*** |
|  |  | **HSD** | **0.19** | **0.11** | **0.66** | **0.23** | **0.26** | **0.15** | **0.30** | **0.08** | **0.41** |

UC-Untreated control, NC-Negative control, OTE-One-time exposure, ME- Multiple exposures

All values are given as Mean±SD

The different small letters (a, b …) within a column are significantly different by Tukey’s HSD (P<0.05)
